# Genetic variability in *ADAM17/TACE* is associated with sporadic Alzheimer’s disease risk, neuropsychiatric symptoms and cognitive performance on the Rey Auditory Verbal Learning and Clock Drawing Tests

**DOI:** 10.1371/journal.pone.0309631

**Published:** 2025-05-06

**Authors:** Francesco Bruno, Mirella A. Aceto, Ersilia Paparazzo, Domenico Arcuri, Francesca Vozzo, Serena Mirante, Beatrice M. Greco, Teresa Serra Cassano, Paolo Abondio, Sonia Canterini, Antonio Malvaso, Alessandro Grecucci, Luigi Citrigno, Silvana Geracitano, Patrizia Spadafora, Gianfranco Puccio, Francesca Frangipane, Sabrina M. Curcio, Francesca Ferrise, Valentina Laganà, Rosanna Colao, Giuseppe Passarino, Amalia C. Bruni, Raffaele Maletta, Francesca Cavalcanti, Alberto Montesanto

**Affiliations:** 1 Department of Human and Social Sciences, Faculty of Social and Communication Sciences, Universitas Mercatorum, Rome, Italy; 2 Department of Biology, Ecology and Earth Sciences, University of Calabria, Rende, Italy; 3 Student at Department of Medical and Surgical Sciences, Magna Graecia University of Catanzaro, Catanzaro, Italy; 4 Student at School of Psychology, University of Florence, Firenze, Italy; 5 Institute for Biomedical Research and Innovation (IRIB), Italian National Research Council (CNR), Mangone, Italy; 6 IRCCS Istituto delle Scienze Neurologiche di Bologna, Bologna, Italy; 7 Division of Neuroscience, Dept. of Psychology, University La Sapienza, Rome, Italy; 8 European Center for Brain Research, IRCCS Fondazione Santa Lucia, Rome, Italy; 9 Neurology Resident at Department of Brain and Behavioral Sciences, University of Pavia, Pavia, Italy; 10 Department of Psychology and Cognitive Sciences, University of Trento, Trento, Italy; 11 Regional Neurogenetic Centre (CRN), Department of Primary Care, Azienda Sanitaria Provinciale Di Catanzaro, Lamezia Terme, CZ, Italy; Sungkyunkwan University - Suwon Campus: Sungkyunkwan University - Natural Sciences Campus, KOREA, REPUBLIC OF

## Abstract

Recent studies have highlighted the significant role of ADAM17/TACE (encoded by *ADAM17/TACE*) in the pathogenesis of Alzheimer’s disease (AD). Yet, the relationship between *ADAM17/TACE* gene polymorphisms and AD was less studied. This study aims to analyse the relationship of *ADAM17/TACE* gene polymorphism with the risk, age of onset, neuropsychiatric manifestations, cognitive impairment, and medial temporal lobe atrophy in sporadic AD (sAD). This case–control association study was conducted in an Italian cohort consisting of 297 sAD patients and 316 controls. Seven tag-SNPs were selected and genotyped. Linear and logistic regression analyses were used to assess the association between parameters of interest and the genetic variability of *ADAM17/TACE*. After Bonferroni correction, our findings underscore the complexity of genetic influences of *ADAM17/TACE* on sAD, particularly the roles of rs12692385 in modulating sAD risk and the performance on the Rey Auditory Verbal Learning Test – delayed recall. In addition, rs13008101 significantly affected the performance on the Clock Drawing Test. Moreover, rs10179642 and rs35280016 were associated with a higher frequency and severity of hallucinations and agitation/aggression, respectively. These results contribute to a deeper understanding of the genetic underpinnings of sAD and may be useful for examining the risk of developing sAD, assessing cognitive deficits, neuropsychiatric symptoms, and informing new therapeutic strategies and future research targeting *ADAM17/TACE*.

Alzheimer’s disease (AD) is the most widespread neurodegenerative disorder affecting more than 24 million people worldwide [1]. Primarily it is characterized by amyloid plaques and neurofibrillary tangles [2]. Amyloid plaques consist of extracellular deposits mostly formed by amyloid-beta (Aβ) peptides, which are produced through the amyloidogenic pathway [3]. In this pathway, amyloid precursor protein (APP) is cleaved by β-secretase and γ-secretase, resulting in Aβ fragments that aggregate into plaques [3,4]. Alternatively, in the non-amyloidogenic pathway, APP is cleaved by α-secretase, producing a non-toxic fragment that prevents the formation of Aβ plaques [4]. Neurofibrillary tangles, composed essentially of hyperphosphorylated *tau* protein, disrupt intracellular transport, contributing to neuronal dysfunction and cognitive decline [5]. Amyloid plaques and neurofibrillary tangles are associated with neuronal and synaptic loss, microglial activation, astrocyte reactivity, and neuroinflammation [6]. Medial temporal lobe atrophy is a hallmark feature of AD and is closely associated with the early and progressive memory impairment characteristic of the disease [7]. Beyond memory impairments, patients often experience difficulties in executive functions, attention, language, and visual-spatial abilities [8–10]. AD is also marked by significant behavioral and psychological symptoms, collectively known as neuropsychiatric symptoms. These include mood disturbances like depression and anxiety, along with agitation/aggression and hallucinations [11,12].

Genetic determinants are known to be involved in AD. Indeed, AD can be caused by mutations in Presenilin 1 (*PS1*), 2 (*PS2*) or Amyloid Precursors Protein (*APP*) genes giving rise to autosomal dominant familial AD (fAD) [6,13–16]. However, in most AD cases, environmental and genetic risk factors may interact causing the onset of sporadic forms (sAD) [17–19]. According to Zang et al. [20], the main environmental factors that significantly influence the risk of sAD include sedentary lifestyles, poor dietary choices, air pollution, socioeconomic status, chronic stress, social isolation, diabetes, and hypertension. Among the genetic factors, the apolipoprotein E (*APOE*) gene, particularly the ε4 allele, is recognized as the principal risk factor for sAD, associated with increased susceptibility to the disease [21]. Recent studies have advanced our understanding of the polygenic nature of sAD, highlighting the role of common genetic variants, including APOE and the Disintegrin and Metalloprotease (ADAM) 10, in risk stratification through polygenic risk scores [22]. Despite the functional overlap between ADAM10 and other members of the ADAM family, such as ADAM17 [23,24], the role of the latter in the genetics of sAD remains poorly characterized.

ADAM17 - also known as Tumor Necrosis Factor-Alpha Converting Enzyme (TACE) - is a crucial sheddase involved in the proteolytic cleavage of over 80 substrates, including cytokines (e.g., TNFα, IL-1R, IL6R), growth factors (e.g., TGFα, EGF), chemokines (e.g., IL-8R), and cell adhesion molecules (CAMs), which are essential for various physiological processes [25]. One of the key functions of ADAM17/TACE, as well as ADAM10, is its involvement in the proteolysis of the APP as an α-secretase [26]. This process prevents the formation of Aβ peptides, which aggregate to form the Aβ plaques typically observed in the brains of AD patients [14]. In addition to its role in APP processing, ADAM17/TACE is also critical in microglial activation, as it mediates the shedding of various inflammatory factors and their receptors, such as TNFα, IL-1R, and IL6R, thereby contributing to the neuroinflammatory environment associated with AD [26,27]. The involvement of ADAM17/TACE in AD has been further substantiated by findings that show increased ADAM17/TACE activity in the cerebrospinal fluid (CSF) of AD patients [26]. This elevated activity correlates with higher levels of soluble TNF receptors, which are proteolytically cleaved by ADAM17/TACE, suggesting a link between the activity of this sheddase and the inflammatory response in AD. More recently, Tian et al. [28] demonstrated that ADAM17/TACE plays a critical role in modulating Aβ levels, neuroinflammation, blood-brain barrier integrity and cognitive functions (i.e., memory performance) in the APP/PS1 mouse model of AD, making it a potential target for novel therapeutic interventions in AD. Despite the importance of ADAM17/TACE in these processes, the genetic factors influencing ADAM17/TACE’s function in AD remain poorly understood. While some studies have not found significant associations between single nucleotide polymorphisms (SNPs) in the *ADAM17/TACE* promoter and sAD risk [29], others have identified specific *ADAM17/TACE* variants that segregate with fAD cases lacking mutations in other common AD-related genes like *APP*, *PS1*, and *PS2* [30]. Given the multifaceted role of ADAM17/TACE in APP processing and neuroinflammation, further research is needed to explore its genetic variations and their potential impact on AD risk, age of onset, clinical manifestations and brain pathology. Considering these premises, the aims of this study were to: 1) select a set of informative SNPs (tag-SNPs) to capture most of the genetic variations of the entire *ADAM17/TACE* gene for the European population; ii) analyze the association between the selected tag-SNPs and the risk and age of onset of sAD, the medial temporal lobe atrophy, the cognitive impairment and neuropsychiatric manifestations in sAD.

## 1. Materials and methods

### 1.1 Study population

The case group includes 297 sAD patients diagnosed and followed at the Regional Neurogenetic Centre (ASP CZ) from 1996 to 2018. The control group includes 316 controls recruited from the structure during the same period (Table 1). All the control subjects were carefully assessed based on their personal and family clinical history and underwent clinical and neurological examinations, to exclude the presence of neurological disorders. None of the control subjects had any family history of AD. For the case group, inclusion criteria were: 1) Diagnosis of probable sAD according to the NINCDS-ADRDA criteria and National Institute on Aging and Alzheimer’s Association Workgroup [31,32]; 2) absence of mutations in *APP*, *PS1,* and *PS2* genes; 3) known *APOE* genotype; 4) availability of a DNA sample; 5) completeness of clinical data. Exclusion criteria were: 1) a concomitant neurologic disorder; (2) severe cardiac, pulmonary, hepatic, renal diseases or any kind of tumor; 3) a history of mental disorders. At the time of diagnosis, all patients underwent the following examinations: a) Mini-Mental State Examination (MMSE) [33,34] to assess general cognitive functioning; b) Clinical Dementia Rating Scale (CDR) [35] to evaluate the dementia severity; c) Clinical Insight Rating Scale (CIRS) [36] to examine the awareness of the disease; d) Activities of Daily Living (ADL) [37] and Instrumental Activities of Daily Living (IADL) [38] to evaluate basic and instrumental functional independence, respectively; e) Neuropsychiatric Inventory (NPI) [39]. The NPI assesses 12 main symptoms: delusions, hallucinations, agitation/aggression, depression/dysphoria, anxiety, euphoria/elation, apathy/indifference, disinhibition, irritability/emotional lability, aberrant motor behavior, sleep and nighttime disturbances, appetite, and eating disturbances. Each symptom is rated by frequency and severity to obtain a total score [39].

**Table 1:** Characteristics of the sAD and control groups.

	sAD (n = 297)	Controls (n = 316)	P-value
Age, years	72.3 ± 8.9	60.4 ± 17.7	<0.001
Age at onset, years	68.3 ± 9.6		
Female, n (%)	180 (61.2)	114 (38.8)	0.002
MMSE	15.8 ± 5.4	–	
CIRS	2.6 ± 3	–	
CDR, n (%)			
Mild (0.5/1)	153 (56.04)		
Moderate (2)	79 (28.94)		
Severe (3/4)	41 (15.02)		
ADL	4.7 ± 1.6	–	
IADL	2.8 ± 2.2	–	
NPI Total Score	10.8 ± 12.9	–	
APOE ε4, n (%)			<0.001
0	165 (56.1)	263 (84.0)	
1	105 (35.7)	46 (14.7)	
2	24 (8.2)	4 (1.3)	

***Note****.* Data are presented as (%) or mean ± standard deviation (SD). MMSE: Mini-Mental State Examination; ADL: Activity of Daily Living; IADL: Instrumental Activity of Daily Living; CDR: Clinical Dementia Rating Scale; CIRS: Clinical Insight Rating Scale; NPI: Neuropsychiatric Inventory. APOE: 0 = no carriers of ε4 allele; 1 = carriers of one e4 allele; 2 = carriers of two e4 alleles.

At the time of diagnosis, 158 sAD patients underwent an extensive neuropsychological assessment which included the administration of: i) the Clock Drawing Test, to assess multiple aspects of cognition, including visuo-constructive and visuo-spatial skills, symbolic and conceptual representation, hemispatial attention, semantic memory, and executive functions such as organization, planning, and parallel processing [40,41]; ii) the Rey Auditory Verbal Learning Test, to evaluate various aspects of verbal memory, including immediate recall, learning efficiency and delayed recall [42]; iii) the Babcock Story Recall Test, to assess verbal memory, particularly episodic memory [41]; iv) the Rey-Osterrieth complex figure copying test, to evaluate visuospatial abilities (e.g., perceptual organization, spatial perception and constructional praxis [43]; v) the Attentive Matrices, to assess key components of attention, including selective attention, sustained attention, and processing speed [44]; vi) the Verbal Fluency Test, to evaluate the ability of language production, executive function, and semantic memory [41]. At the same time, 56 sAD patients underwent brain MRI scans that were analyzed by a trained neurologist for extracting the rating scores of *Medial Temporal Lobe Atrophy* (MTA; to assess the degree of atrophy in the hippocampus, parahippocampal gyrus, and surrounding CSF space – ranging from 0 = ‘no atrophy’ to 4 = ‘severe loss of hippocampal volume’) [45]. The work was done according to the Helsinki Declaration of 1975. The study protocol was approved by the regional Ethical Committee, Catanzaro, Italy (Prot. CET 77/2023). Written consent for genetic screening was obtained from all participants or, where appropriate, by their relatives or legal representatives.

### 1.2 Tag-SNP selection and genotyping

The gene encoding *ADAM17/TACE* is located in the region 2p25.1 between positions 9,628,615 and 9,695,921 (GRCh37 genome build). The sequence identified by GenBank and analysed here includes 1 kb upstream of the transcription start site, 3 kb downstream of the stop codon, 19 exons, and 18 introns. Using the data from the 1000 genomes project and referring to 503 individuals of European descent (CEU, STI, GBR, FIN, IBS), 523 SNPs were identified within this sequence. The linkage disequilibrium (LD) profile region was analyzed using the solid spine of LD algorithm as implemented in Haploview (v4.2) [46]. Tag-SNPs were identified using Haploview Tagger Program (http://www.broad.mit.edu/mpg/tagger/) with a r^2^ of 0.8. Genetic profiling was carried out on the DNA extracted from blood samples provided at the recruitment visit. Genotyping of selected tag-SNP was performed by QuantStudio^TM^ 3 Real-Time PCR System using TaqMan genotyping assays following the manufacturer’s instruction and 10 ng of DNA mixed with the TaqMan Genotyping Master Mix (Thermo Fisher Scientific).

### 1.3 Statistical analyses

Allele frequencies for each SNP were determined by gene counting. The genotype distribution of each SNP was tested for deviation from Hardy-Weinberg Equilibrium (HWE) in controls by using Fisher’s exact test. Demographic, clinical, and biological data were summarized with counts and percentages for categorical variables and means (standard deviations) for continuous variables. Linear and logistic regression analyses were used to assess the association between parameters of interest and the genetic variability of *ADAM17/TACE*. In particular, logistic regression analyses were used to estimate how the variability of *ADAM17/TACE* influences the predisposition to sAD. Analyses were corrected for age, sex, and APOE status (number of APOE ε4 alleles). Associations were presented as odds ratios (OR) and their corresponding Confidence Intervals (CI). Linear regression models were used to estimate how the variability of *ADAM17/TACE* influences the age onset, NPI score, and cognitive performance in sAD. For standardizing the distribution, values were transformed into z-scores, by subtracting the mean and dividing by the standard deviation for all neuropsychological tests. MTA score (0: no atrophy; 1: from mild to severe atrophy) was used as the response variable in the frame of a logistic regression model to test how the variability of *ADAM17/TACE* influences MRI measurements. Data were analysed using R (version 4.4.0). P-values < 0.05 were deemed as nominally significant. The nominal p-values obtained in the genetic association analyses have been adjusted for multiple comparisons using the Bonferroni correction (0.05/7 SNPs = 0.007).

## 2. Results

### 2.1 Characteristics of the study population

Among the 297 sAD patients and 316 controls recruited, controls were around 10 years younger than cases, while cases showed a higher prevalence of women. For this reason, age and gender were considered as potential confounding variables in the following association analyses. As expected, more APOE ε4-carriers were found in the sAD group (p < 0.001). The detailed characteristics of the study population are shown in Table 1.

### 2.2 Construction of LD blocks and selection of tag-SNPs

**Fig 1:**
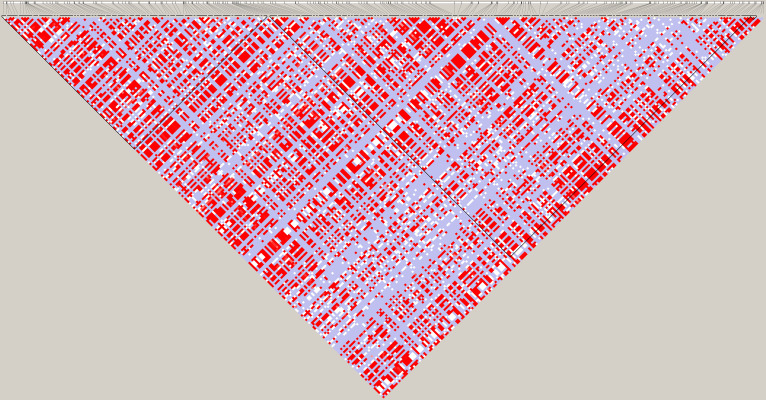
Linkage disequilibrium (LD) profile and block partition of the *ADAM17/TACE* gene. Pairwise LD coefficients R-squared×100 are shown in each cell. The standard color scheme of Haploview was used for the LD color display.

125 of 523 *ADAM17/TACE* polymorphisms with a minor allele frequency (MAF) >10% analysed defined 3 blocks of LD (Fig 1).

We identified 7 SNPs that tagged the full set of 118 SNPs with a MAF > 0.10 and pairwise correlation (R^2^) > 0.8, whose characteristics are reported in Table 2. The genotype distribution of all selected tag-SNPs was in agreement with Hardy-Weinberg equilibrium (p > 0.05).

**Table 2:** The selected tag-SNPs.

tag-SNP	Location	Allele (major/minor)	CR	MAF in sAD	MAF in controls	P-value (HWE)^*^
rs13008101	3’ UTR	G/T	97.1	46.2	45.8	0.730
rs11690078	Intron 13	T/C	97.7	40.1	38.4	0.630
rs35280016	Intron 13	G/A	95.8	19.6	17.8	0.564
rs55694483	Intron 10	A/G	94.6	46.4	43.7	1.000
rs12464398	Intron 4	T/C	97.6	31.2	33.5	0.306
rs10179642	Intron 1	T/C	98.0	13.7	17.7	0.434
rs12692385	Intron 1	T/C	97.4	34.3	26.1	0.768

***Note****.* MAF: Minor Allele Frequency (%); CR: Call Rate. HWE: Hardy-Weinberg Equilibrium.

*HWE tested in controls

### 2.3 Association between tag-SNPs within the ADAM17/TACE gene and the risk of sAD

As shown in Table 3, after adjustments for age, sex, and *APOE* status, rs12692385 C-allele carriers have a significantly increased risk of developing sAD (dominant model, OR = 1.59, 95% CI = 1.13–2.24, *p* = 0.008; additive model, OR = 1.45, 95% CI = 1.11–1.89, *p* = 0.006). However, only the additive model remains significant after the Bonferroni correction. No significant associations were found between the other tag-SNPs and the risk of sAD (S1 Table).

**Table 3:** Genotype distributions and association of the rs12692385 with the risk of sAD.

Genotypes	Control group	sAD group	Genetic model					
			Additive		Dominant		Recessive	
			OR (95% CI)	P-value	OR (95% CI)	P-value	OR (95% CI)	P-value
T/T	55.0%	42.8%	1.45 (1.11–1.89)	**0.006**	1.59 (1.13–2.24)	**0.008**	1.63 (0.91–2.98)	0.106
C/T	37.8%	45.3%						
C/C	7.2%	11.9%						

### 2.4 Association between tag-SNPs within the ADAM17/TACE gene and the age of onset of sAD

After adjustments for age, sex, and *APOE* status, rs13008101 was nominally associated with a delay of age onset in sAD (dominant model, b = 2.82, 95% CI = 0.36–5.27, *p* = 0.025). T-allele carriers have an average age of onset 2.82 years later than homozygotes G/G subjects. However, this association was not retained after the Bonferroni correction. No significant associations were found between the other tag-SNPs and the age of onset (S2 Table).

### 2.5 Association between tag-SNPs within the ADAM17/TACE gene and the medial temporal atrophy in sAD

rs35280016 was nominally associated with a severe loss of hippocampal volume in sAD patients (dominant model, OR = 0.1, 95% CI = 0.00–0.72, *p* = 0.046), indicating that A-allele carriers showed a lower atrophy than homozygotes G/G subjects. However, this association was not retained after the Bonferroni correction. No significant associations were found between the other tag-SNPs and the medial temporal lobe atrophy (S3 Table).

### 2.6 Association between tag-SNPs within the ADAM17/TACE gene and the cognitive impairment in sAD

rs12464398 was nominally associated with the Rey Auditory Verbal Learning Test – immediate recall score (dominant model, b = -0.45, 95% CI = -0.90 – -0.010, *p* = 0.047; additive model, b = -0.24, 95% CI = -0.46 – -0.02, *p* = 0.036); T-allele carriers had worse performance on this test compared to homozygotes C/C subjects. However, this association was not retained after the Bonferroni correction (S4 Table). The rs12464398 (dominant model, b = -0.5, 95% CI = -0.98 – -0.02, *p* = 0.041), rs12692385 (dominant model, b = -0.76, 95% CI = -1.28 – -0.24, *p* = 0.004), rs13008101 (additive model, b = -0.26, 95% CI = -0.49 – -0.03, *p* = 0.027) were nominally associated with the Rey Auditory Verbal Learning Test– delayed recall score with T-allele carriers for all three SNPs showing a lower performance on this test compared to remaining genotypes. However, only the association with rs12692385 remained significant after the Bonferroni correction (Table 4, S5 Table).

**Table 4:** Genotype distributions and associations of rs12692385 with the Rey Auditory Verbal Learning Test – delayed recall score.

Genotypes	sAD group	Genetic model					
		Additive		Dominant		Recessive	
		Mean difference (95% CI)	P-value	Mean difference (95% CI)	P-value	Mean difference (95% CI)	P-value
T/T	43.3%	-0.17 (-0.42–0.09)	0.196	-0.76 (-1.28 – -0.24)	**0.004**	0.00 (-0.33–0.34)	0.986
C/T	46.1%						
C/C	10.6%						

**Fig 2:**
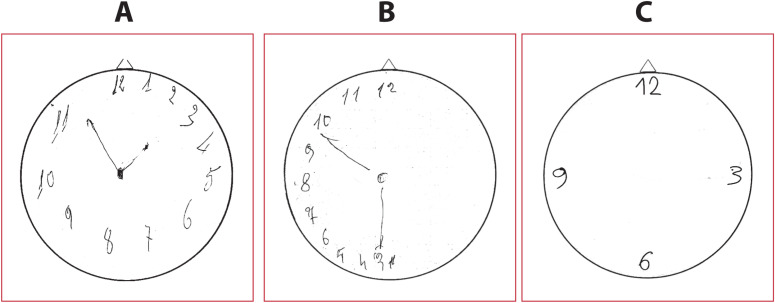
Performance on Clock Drawing Test based on rs13008101 genotype. (A) G/G patients make minor errors in the arrangement of numbers and/or in the clock hands placement; (B) T/G carriers show a more pronounced deficit; (C) T/T patients are unable to report the numbers and clock hands even if the experimenter marks some numbers, as illustrated in the example given.

Both rs35280016 (additive model, b = 0.32, 95% CI = 0.01–0.62, *p* = 0.043) and rs55694483 (additive model, b = 0.27, 95% CI = 0.03–0.51, *p* = 0.026) were nominally associated with a significant effect on the Babcock Story Recall Test score, G-allele carriers showing a better performance to this test compared to homozygotes A/A subjects. However, these associations did not retain after the Bonferroni correction (S6 Table). Similarly, rs13008101 was nominally associated with a significant effect on Clock Drawing Test score with T-allele carriers having a lower score with respect to G-allele carriers (dominant model, b = -0.76, 95% CI = -1.21 – -0.32, *p* = 0.001; additive model, b = -0.45, 95% CI = -0.76 – -0.15, *p* = 0.003) (Fig 2). This association remains significant after Bonferroni correction (Table 5). No significant associations were found between the other tag-SNPs and the performance on the Clock Drawing Test (S7 Table).

**Table 5:** Genotype distributions and association of rs13008101 with the Clock Drawing Test score.

Genotypes	sAD group	Genetic model					
		Additive		Dominant		Recessive	
		Mean difference (95% CI)	P-value	Mean difference (95% CI)	P-value	Mean difference (95% CI)	P-value
G/G	27.6%	-0.45 (-0.76 – -0.15)	**0.003**	-0.76 (-1.21 – -0.32)	**0.001**	-0.35 (-0.90–0.19)	0.206
T/G	54.0%						
T/T	18.4%						

Finally, rs11690078-T was nominally associated with worse performance on the Rey-Osterrieth complex figure copying test compared to homozygotes C/C subjects (recessive model, b = -0.67, 95% CI = -1.20 – -0.14, *p* = 0.013; additive model, b = -0.38, 95% CI = -0.75 – -0.02, *p* = 0.039). However, this association was not retained after the Bonferroni correction (S8 Table). No significant associations were found between the other tag-SNPs and the Attentive Matrices and Verbal Fluency Test scores (S9 and S10 Tables).

### 2.7 Association between tag-SNPs within the ADAM17/TACE gene and the neuropsychiatric manifestations in sAD

rs13008101 was nominally associated with a lower NPI total score (recessive model, b = -4.47, 95% CI = -8.22 – -0.71, *p* = 0.02), indicating that homozygotes T/T subjects manifest less neuropsychiatric symptoms compared to G-allele carriers. In the same manner, this SNP result associated with: i) NPI-delusions (dominant model, b = 0.50, 95% CI = 0.01–0.99, p = 0.045) - with T-allele carriers had a higher NPI-delusions score concerning homozygotes G/G subjects; ii) NPI - appetite/eating disturbances (recessive model, b = -0.73, 95% CI = -1.42 – -0.03, p = 0.040), indicating that the T/T homozygotes had lower scores compared to G/G and T/G carriers; iii) NPI- sleep and nighttime disturbances (recessive model, b = -1.04, 95% CI = -1.85 – -0.24, p = 0.011) indicating that the T/T homozygotes had lower scores compared to the other genotypes. rs10179642 was nominally associated with a higher NPI-hallucinations score (recessive model, b = 3.76, 95% CI = 2.33–5.18, *p* < 0.001), indicating that homozygotes C/C subjects manifest higher frequency and severity of hallucinations compared to G-allele carriers. rs35280016-A variant was nominally associated with a higher NPI – agitation/aggression score (recessive model, b = 3.61, 95% CI = 1.70–5.53, *p* < 0.001; additive model, b = 0.85, 95% CI = 0.14–1.55, p = 0.018), indicating that allele A carriers manifest higher frequency and severity of agitation/aggression compared to the remaining genotypes. rs12464398 was nominally associated with a higher NPI-irritability score (dominant model, b = 0.82, 95% CI = 0.03–1.61, p = 0.042), indicating that to homozygotes C-allele carriers manifest higher frequency and severity of irritability compared to T/T subjects. rs10179642 was nominally associated with a higher NPI-depression score (dominant model, b = 0.76, 95% CI = 0.02–1.51, p = 0.045), indicating that to homozygotes C-allele carriers manifest higher frequency and severity of depression compared to T/T subjects. rs55694483 was nominally associated with a higher NPI-euphoria score (recessive model, b = 0.13, 95% CI = 0.00–0.25, p = 0.047), indicating that G/G patients manifest higher frequency and severity of euphoria compared to the remaining genotypes. No significant associations were found between the other tag-SNPs and NPI total score and subscores. However, only the association between rs10179642 with hallucinations (recessive model) and rs35280016 and agitation/aggression (recessive model) remain significant after Bonferroni correction (Table 6, S11 and S12 Tables).

**Table 6:** Genotype distributions and association of rs10179642 and rs35280016 with NPI subscores.

	Genotypes	sAD group	Genetic model					
			Additive		Dominant		Recessive	
			Mean difference (95% CI)	P-value	Mean difference (95% CI)	P-value	Mean difference (95% CI)	P-value
			**Hallucinations**					
rs10179642	T/T	73.4%	0.34 (-0.03–0.71)	0.072	0.13 (-0.28–0.53)	0.536	3.76 (2.33–5.18)	**<0.001**
	C/T	25.2%						
	C/C	1.4%						
			**Agitation/Aggression**					
rs35280016	G/G	65.0	0.85 (0.14–1.55)	**0.018**	0.54 (-0.31–1.40)	0.212	3.61 (1.70–5.53)	**<0.001**
	A/G	30.5						
	A/A	4.5						

## 3. Discussion

To our knowledge, this study represents the first attempt to select and test the associations between *ADAM17/TACE* tag-SNPs, representative of the entire gene for the European population, and the risk, age of onset, neuropsychiatric manifestations, cognitive impairment, and medial temporal lobe atrophy in sAD patients. The analysis carried out allowed the identification of 7 tag-SNPs: rs13008101 (3’ UTR), rs11690078 (Intron 13), rs35280016 (Intron 13), rs55694483 (Intron 10), rs12464398 (Intron 4), rs10179642 (Intron 1), rs12692385 (Intron 1).

Our study found that the rs12692385-C variant is associated with an increased risk of sAD in the European population. In contrast, Wang et al. [29] reported no association between the rs12692386 polymorphism, located in the same haplotype block, and sAD risk in a Northern Chinese Han population. These differences may be attributed to the distinct ethnic backgrounds of the study participants, emphasizing the importance of conducting population-specific genetic research. Furthermore, our findings suggest that incorporating the rs12692385 variant into genetic screening could be pivotal for identifying European individuals at elevated risk of developing sAD. This approach may enhance early detection and enable personalized interventions, particularly within specific population contexts. Identifying individuals with a higher genetic predisposition would allow for more effective preventive measures and closer monitoring, both of which are critical for mitigating disease progression. The role of this polymorphism in sAD is further supported by our finding of an association between the rs12692385-T allele and poorer performance on the delayed recall component of the Rey Auditory Verbal Learning Test. The Rey Auditory Verbal Learning Test, a well-established measure of verbal memory, involves recalling a 15-word list presented audibly multiple times and assesses various memory aspects [42]. Studies have shown that damage to areas like the temporal lobe and hippocampus, which are critical in AD, often leads to difficulties in delayed recall [47–50]. Such impairments on the Rey Auditory Verbal Learning Test serve as early indicators of AD and can help identify individuals likely to progress from memory complaints to the disorder [51,52]. Given the prominence of verbal memory deficits in AD, understanding the genetic influences behind these impairments may enhance early diagnosis and intervention strategies. In summary, our results demonstrate evidence of the involvement of rs12692385 in both the sAD pathogenesis and verbal memory dysfunction, highlighting its potential relevance in early diagnosis and risk assessment.

Furthermore, we found an association between the T-allele of rs13008101 and poorer performance on both the Clock Drawing Test. The Clock Drawing Test is a widely used neuropsychological assessment tool designed to evaluate several aspects of cognitive functioning, including visuo-constructive and visuo-spatial skills, symbolic and conceptual representation, hemispatial attention, semantic memory, and executive functions such as organization, planning, and parallel processing [40,41]. Typically, patients are shown a blank circle and asked to fill in the numbers of a clock face and set the hands to a specified time (i.e., “11:10”) [41]. In the context of AD, the CDT serves as a valuable tool for early detection and monitoring of cognitive decline. Research indicates that individuals with AD often exhibit various difficulties with the task, leading to errors in spatial arrangement, numbers placement, and clock hands positioning [53,54]. Interestingly, we observed that performances varied according to the alleles of the rs13008101 polymorphism, where G is the major allele. Individuals with the G/G genotype tended to make minor errors in the arrangement of numbers and/or clock hands. In contrast, those with the T/G genotype showed greater impairments, whereas individuals with the T/T genotype struggled to complete the task (Fig 2). These findings strengthen the discriminative power of this SNP concerning clock drawing performance, reinforcing a potential genetic influence of *ADAM17/TACE* on cognitive function in sAD.

Finally, our results revealed that rs10179642 and rs35280016 were associated with a higher frequency and severity of hallucinations and agitation/aggression, respectively. These neuropsychiatric symptoms are also observed in other neurodegenerative diseases, such as frontotemporal dementia (FTD), where behavioral and cognitive changes dominate the clinical picture [11,55,56]. In FTD, neuroinflammation, microgliosis, and neuronal loss are well-documented [57], similar to the pathological hallmarks of AD [6]. However, while amyloid plaques and neurofibrillary tangles characterize AD, FTD is typically associated with tauopathies or TDP-43 protein accumulation [58]. The shared neuroinflammatory responses between these conditions, such as microgliosis and astrogliosis [6,57], could contribute to symptom overlap, making early differential diagnosis challenging [59]. Given the significant associations of rs10179642 and rs35280016 with hallucinations and agitation/aggression in our sAD cohort, these polymorphisms might serve as valuable biomarkers for distinguishing sAD from FTD, especially in early disease phases when clinical features can be ambiguous. However, further research is required to explore these polymorphisms’ role in FTD, particularly regarding their impact in the early stages of the disease.

Single nucleotide variants rs12692385, rs13008101, rs12464398 and rs10179642 have also reported the most interesting results when checked on the GTEx database (https://gtexportal.org/home/), RegulomeDB (https://www.regulomedb.org/regulome-search/) and FORGEdb (https://forgedb.cancer.gov/) to infer their functional impact. Indeed, the first two variants emerge as eQTLs (expression quantitative trait loci) with high significance in the brain cortex (p-values = 2.1e-14 and 4.2e-11, respectively), while the first three are relevant in the cerebellum (p-values = 2.2e-11, 2.5e-10 and 9.3e-9, respectively), with rs12692385 being also significantly involved in modulating expression in the caudate (basal ganglia; p-value = 3.2e-10) and the hypothalamus (p-value = 7.3e-8), while rs10179642 does not show brain specificity for an eQTL function. As far as RegulomeDB ranks, rs12692385, rs12464398 and rs10179642 are assigned a rank of 1f and a score of 0.553–0.554, indicating that the eQTL function is recognized (in various tissue samples, but not only in the brain to not necessarily in the brain) together with transcription factor binding prediction; rs13008101 is assigned a rank of 1b and a score of 0.94, suggesting a much stronger confirmation of expression regulation, transcription factor motif and DNase footprint. Finally, FORGEdb, which condenses several characteristics to determine a prediction of regulatory function, assigns scores for the variants rs12692385, rs13008101, rs12464398, and rs10179642 respectively 6, 9, 4 and 6 on a scale of 1–10, suggesting that both these variants are influential in terms of modulation of transcription and gene expression, even though the last variant appears to have a weaker involvement in the brain when compared to the other three. Overall, it seems likely that rs13008101 has the highest involvement in modulating the regulation of transcription in the brain.

There are some limitations to our work. First, the limited small size might affect the generalizability of the findings. For this reason, it is essential to replicate the reported results in a larger sample and, possibly, in other countries. However, we emphasize that both the patient and control groups were collected from Calabria in Southern Italy, a region known for its high genetic homogeneity due to historical and geographical isolation until recent times [60,61]. Moreover, it is worth noting that we provide in silico evidence supporting the hypothesis that the detrimental/beneficial effects of the investigated variants may depend on the increased/decreased expression of *ADAM17/TACE*. In addition, since we adopted a tagging approach, there is a reasonable chance that the SNPs we identified as significantly associated with AD and the phenotypes under investigation may not directly identify the direct susceptibility variant, but the result of their correlation (LD) with the unmeasured flanking SNPs. Although our study does not allow definitive conclusions, to the best of our knowledge, it represents the first work that investigated the role of the genetic variability of *ADAM17/TACE* on the susceptibility to sAD, providing novel insights and future directions aimed at unrevealing the complex genetic architecture of sAD.

## 4. Conclusions

In summary, our study underscores the complexity of genetic influences on sAD, particularly the roles of various *ADAM17/TACE* gene variants in modulating disease risk, cognitive impairment, and neuropsychiatric symptoms. These findings suggest that genetic markers like rs12692385, rs13008101, rs10179642, and rs35280016 could provide valuable insights into disease mechanisms and help guide personalized diagnostic and therapeutic strategies. Future research should focus on elucidating the molecular mechanisms by which these SNPs affect ADAM17/TACE function and exploring their potential as targets for modifying disease progression and cognitive decline.

## Supporting information

S1 Table Genotype distributions of the other six tag-SNPs and their associations with the risk of sAD. (DOCX)

S2 Table Genotype distributions of the tag-SNPs and their associations with the age of onset. (DOCX)

S3 Table Genotype distributions of the tag-SNPs and their associations with the medial temporal lobe atrophy. (DOCX)

S4 Table Genotype distributions of the tag-SNPs and their associations with the Rey Auditory Verbal Learning Test – immediate recall score. (DOCX)

S5 Table Genotype distributions of the tag-SNPs and their associations with the Rey Auditory Verbal Learning Test – delayed recall score. (DOCX)

S6 Table Genotype distributions of the tag-SNPs and their associations with the Babcock Story Recall Test score. (DOCX)

S7 Table Genotype distributions of the tag-SNPs and their associations with the Clock Drawing Test score. (DOCX)

S8 Table Genotype distributions of the tag-SNPs and their associations with the Rey-Osterrieth complex figure copying test score. (DOCX)

S9 Table Genotype distributions of the tag-SNPs and their associations with the Attentive Matrices score. (DOCX)

S10 Table Genotype distributions of the tag-SNPs and their associations with the Verbal Fluency Test score. (DOCX)

S11 Table Genotype distributions of the tag-SNPs and their associations with the NPI Total Score. (DOCX)

S12 Table Genotype distributions of the tag-SNPs showing a nominal association with NPI subscores. (DOCX)
