# Supplementary material for: Genetic variability in ADAM17/TACE is associated with sporadic Alzheimer’s disease risk, neuropsychiatric symptoms and cognitive performance on the Rey Auditory Verbal Learning and Clock Drawing Tests
Source: PLoS One. 2025 May 6;20(5):e0309631. doi: 10.1371/journal.pone.0309631 (PMC12054869; doi:10.1371/journal.pone.0309631)
Supplement: S1 Table — (DOCX) [file pone.0309631.s001.docx]

**S1 Table. Genotype distributions of the other six tag-SNPs and their associations with the risk of sAD**

| **Tag-SNPs** | **Genotypes** | **Control group** | **sAD group** | **Genetic model** | | | | | |
| --- | --- | --- | --- | --- | --- | --- | --- | --- | --- |
|  |  |  |  | **Additive** | | **Dominant** | | **Recessive** | |
|  |  |  |  | **OR (95% CI)** | **P-value** | **OR (95% CI)** | **P-value** | **OR (95% CI)** | **P-value** |
| **rs11690078** | T/T | 37.40% | 35.80% | 1.06 (0.83 – 1.37) | 0.623 | 1.08 (0.76 – 1.54) | 0.664 | 1.09 (0.68 – 1.76) | 0.72 |
|  | C/T | 48.50% | 47.50% |  |  |  |  |  |  |
|  | C/C | 14.10% | 16.70% |  |  |  |  |  |  |
| **rs35280016** | G/G | 66.90% | 65.30% | 1.06 (0.77 – 1.45) | 0.715 | 1.03 (0.72 – 1.48) | 0.874 | 1.41 (0.54 – 3.88) | 0.487 |
|  | A/G | 30.50% | 30.30% |  |  |  |  |  |  |
|  | A/A | 2.60% | 4.40% |  |  |  |  |  |  |
| **rs55694483** | A/A | 32% | 27.90% | 1.08 (0.84 – 1.38) | 0.554 | 1.14 (0.78 – 1.67) | 0.49 | 1.06 (0.69 – 1.62) | 0.807 |
|  | G/A | 49% | 51.00% |  |  |  |  |  |  |
|  | G/G | 19.30% | 21.40% |  |  |  |  |  |  |
| **rs12464398** | T/T | 45.60% | 50.30% | 0.95 (0.75 – 1.22) | 0.704 | 0.88 (0.63 – 1.24) | 0.468 | 1.08 (0.65 – 1.79) | 0.768 |
|  | T/C | 41.70% | 37.10% |  |  |  |  |  |  |
|  | C/C | 12.70% | 12.60% |  |  |  |  |  |  |
| **rs10179642** | T/T | 68.40% | 74% | 0.74 (0.53 – 1.03) | 0.076 | 0.75 (0.52 – 1.10) | 0.143 | 0.39 (0.10 – 1.18) | 0.118 |
|  | C/T | 27.70% | 24.60% |  |  |  |  |  |  |
|  | C/C | 3.90% | 1.40% |  |  |  |  |  |  |
| **rs13008101** | G/G | 30.20% | 28.40% | 1.04 (0.82 – 1.33) | 0.727 | 1.10 (0.75 – 1.60) | 0.634 | 1.02 (0.67 – 1.54) | 0.939 |
|  | T/G | 48.20% | 50.20% |  |  |  |  |  |  |
|  | T/T | 21.60% | 21.40% |  |  |  |  |  |  |
