# Supplementary material for: Genetic variability in ADAM17/TACE is associated with sporadic Alzheimer’s disease risk, neuropsychiatric symptoms and cognitive performance on the Rey Auditory Verbal Learning and Clock Drawing Tests
Source: PLoS One. 2025 May 6;20(5):e0309631. doi: 10.1371/journal.pone.0309631 (PMC12054869; doi:10.1371/journal.pone.0309631)
Supplement: S2 Table — (DOCX) [file pone.0309631.s002.docx]

**S2 Table.** **Genotype distributions of the tag-SNPs and their associations with the age of onset**

| **Tag-SNPs** | **Genotypes** | **sAD group** | **Genetic model** | | | | | |
| --- | --- | --- | --- | --- | --- | --- | --- | --- |
|  |  |  | **Additive** | | **Dominant** | | **Recessive** | |
|  |  |  | **Mean Difference (95% CI)** | **P-value** | **Mean Difference (95% CI)** | **P-value** | **Mean Difference (95% CI)** | **P-value** |
| **rs11690078** | T/T | 35.88% | -0.17 (-1.75– 1.42) | 0.835 | 0.68 (-1.63 – 2.99) | 0.563 | -1.73 (-4.70– 1.24) | 0.254 |
|  | C/T | 47.40% |  |  |  |  |  |  |
|  | C/C | 16.72% |  |  |  |  |  |  |
| **rs35280016** | G/G | 65.20% | -0.93 (-2.95 – 1.09) | 0.366 | -1.19 (-3.61– 1.23) | 0.334 | -0.78(-6.39 – 4.83) | 0.785 |
|  | A/G | 30.40% |  |  |  |  |  |  |
|  | A/A | 4.40% |  |  |  |  |  |  |
| **rs55694483** | A/A | 28.00% | 0.25(-1.38 – 1.88) | 0.762 | 1.61(-0.92 – 4.14) | 0.213 | -1.2(-3.98 – 1.58) | 0.397 |
|  | G/A | 50.55% |  |  |  |  |  |  |
|  | G/G | 21.45% |  |  |  |  |  |  |
| **rs12464398** | T/T | 50.53% | 1.37(-0.23 – 2.96) | 0.093 | 1.49(-0.74 – 3.71) | 0.191 | 2.66(-0.68 – 6.01) | 0.119 |
|  | T/C | 36.84% |  |  |  |  |  |  |
|  | C/C | 12.63% |  |  |  |  |  |  |
| **rs10179642** | T/T | 73.87% | -0.08(-2.42 – 2.25) | 0.944 | -0.13(-2.66 – 2.41) | 0.922 | 0.39(-9.07 – 9.85) | 0.936 |
|  | C/T | 24.74% |  |  |  |  |  |  |
|  | C/C | 1.39% |  |  |  |  |  |  |
| **rs12692385** | T/T | 42.96% | 0.61(-1.05 – 2.26) | 0.473 | 1.58(-0.67 – 3.82) | 0.169 | -1.07(-4.51 – 2.37) | 0.543 |
|  | C/T | 45.07% |  |  |  |  |  |  |
|  | C/C | 11.97% |  |  |  |  |  |  |
| **rs13008101** | G/G | 28.52% | 1.12(-0.47 – 2.71) | 0.167 | 2.82(0.36 – 5.27) | **0.025** | -0.14(-2.87 – 2.60) | 0.923 |
|  | T/G | 50.00% |  |  |  |  |  |  |
|  | T/T | 21.48% |  |  |  |  |  |  |
