# Supplementary material for: Genetic variability in ADAM17/TACE is associated with sporadic Alzheimer’s disease risk, neuropsychiatric symptoms and cognitive performance on the Rey Auditory Verbal Learning and Clock Drawing Tests
Source: PLoS One. 2025 May 6;20(5):e0309631. doi: 10.1371/journal.pone.0309631 (PMC12054869; doi:10.1371/journal.pone.0309631)
Supplement: S3 Table — (DOCX) [file pone.0309631.s003.docx]

**S3 Table. Genotype distributions of the tag-SNPs and their associations with the medial temporal lobe atrophy**

| **Tag-SNPs** | **Genotypes** | **At Risk (Score 0)** | **At Risk (Score 1-3)** | **Genetic model** | | | | | |
| --- | --- | --- | --- | --- | --- | --- | --- | --- | --- |
|  |  |  |  | **Additive** | | **Dominant** | | **Recessive** | |
|  |  |  |  | **OR (95% CI)** | **P-value** | **OR (95% CI)** | **P-value** | **OR (95% CI)** | **P-value** |
| **rs11690078** | T/T | 85.70% | 44.44% | 6.02 (1.22 – 110.12) | 0.084 | 7.5 (1.15 – 147.85) | 0.072 | - | - |
|  | C/T | 14.30% | 37.78% |  |  |  |  |  |  |
|  | C/C | 0% | 17.78% |  |  |  |  |  |  |
| **rs35280016** | G/G | 16.67% | 66.66% | 0.32 (0.08 – 1.09) | 0.073 | 0.10 (0.00 – 0.72) | **0.046** | 0.50 0.05 – 11.21) | 0.58 |
|  | A/G | 66.66% | 24.24% |  |  |  |  |  |  |
|  | A/A | 16.67% | 9.10% |  |  |  |  |  |  |
| **rs55694483** | A/A | 71.43% | 36.36% | 3.92(1.03 – 26.29) | 0.083 | 4.37(0.84 – 33.09) | 0.099 | - | - |
|  | G/A | 28.57% | 43.19% |  |  |  |  |  |  |
|  | G/G | 0.00% | 20.45% |  |  |  |  |  |  |
| **rs12464398** | T/T | 42.86% | 42.22% | 1.23 (0.46 – 3.76) | 0.690 | 1.03 (0.18 – 5.19) | 0.975 | 2.18 (0.32 – 43.45) | 0.491 |
|  | T/C | 42.86% | 31.12% |  |  |  |  |  |  |
|  | C/C | 14.28% | 26.66% |  |  |  |  |  |  |
| **rs10179642** | T/T | 100.00% | 82.60% | - | - | - | - | - | - |
|  | C/T | 0.00% | 15.22% |  |  |  |  |  |  |
|  | C/C | 0.00% | 2.18% |  |  |  |  |  |  |
| **rs12692385** | T/T | 71.42% | 37.78% | 3.80 (0.96 – 26.11) | 0.098 | 4.12 (0.79 – 31.05) | 0.112 | - | - |
|  | C/T | 28.58% | 46.66% |  |  |  |  |  |  |
|  | C/C | 0.00% | 15.56% |  |  |  |  |  |  |
| **rs13008101** | G/G | 57.14% | 37.20% | 2.45 (0.76 – 11.12) | 0.173 | 2.25 (0.44 – 12.68) | 0.326 | - | - |
|  | T/G | 42.86% | 0.00% |  |  |  |  |  |  |
|  | T/T | 0.00% | 62.80% |  |  |  |  |  |  |
