# Supplementary material for: Genetic variability in ADAM17/TACE is associated with sporadic Alzheimer’s disease risk, neuropsychiatric symptoms and cognitive performance on the Rey Auditory Verbal Learning and Clock Drawing Tests
Source: PLoS One. 2025 May 6;20(5):e0309631. doi: 10.1371/journal.pone.0309631 (PMC12054869; doi:10.1371/journal.pone.0309631)
Supplement: S4 Table — (DOCX) [file pone.0309631.s004.docx]

**S4 Table.** **Genotype distributions of the tag-SNPs and their associations with the Rey Auditory Verbal Learning Test – immediate recall score**

| **Tag-SNPs** | **Genotypes** | **sAD group** | **Genetic model** | | | | | |
| --- | --- | --- | --- | --- | --- | --- | --- | --- |
|  |  |  | **Additive** | | **Dominant** | | **Recessive** | |
|  |  |  | **Mean Difference (95% CI)** | **P-value** | **Mean Difference (95% CI)** | **P-value** | **Mean Difference (95% CI)** | **P-value** |
| **rs11690078** | C/C | 15.82% | 0.12 (-0.10 – 0.35) | 0.279 | 0.37 (-0.05 – 0.80) | 0.086 | 0.04 ( -0.28 – 0.37) | 0.788 |
|  | C/T | 46.84% |  |  |  |  |  |  |
|  | T/T | 37.34% |  |  |  |  |  |  |
| **rs35280016** | A/A | 3.38% | 0.22 (-0.07 – 0.51) | 0.145 | 0.67 (-0.23 – 1.57) | 0.145 | 0.19 (-0.14 – 0.53) | 0.26 |
|  | A/G | 35.14% |  |  |  |  |  |  |
|  | G/G | 61.48% |  |  |  |  |  |  |
| **rs55694483** | A/A | 31.70% | -0.03(-0.27 – 0.21) | 0.796 | 0.11(-0.25 – 0.46) | 0.550 | -0.25(-0.67 – 0.17) | 0.239 |
|  | A/G | 49.30% |  |  |  |  |  |  |
|  | G/G | 19.00% |  |  |  |  |  |  |
| **rs12464398** | C/C | 14.02% | -0.24(-0.46 – -0.02) | **0.036** | -0.45(-0.90 – -0.01) | **0.047** | -0.25(-0.57 – 0.06) | 0.116 |
|  | C/T | 40.13% |  |  |  |  |  |  |
|  | T/T | 45.85% |  |  |  |  |  |  |
| **rs10179642** | C/C | 0.63% | 0.07(-0.28 – 0.42) | 0.681 | -0.13(-2.11 – 1.85) | 0.896 | 0.08(-0.28 – 0.45) | 0.651 |
|  | C/T | 24.05% |  |  |  |  |  |  |
|  | T/T | 75.32% |  |  |  |  |  |  |
| **rs12692385** | C/C | 10.32% | -0.05(-0.29 – 0.19) | 0.677 | 0.03(-0.49 – 0.55) | 0.910 | -0.1(-0.42 – 0.22) | 0.535 |
|  | C/T | 46.46% |  |  |  |  |  |  |
|  | T/T | 43.22% |  |  |  |  |  |  |
| **rs13008101** | G/G | 30.96% | 0(-0.22 – 0.23) | 0.966 | 0.28(-0.06 – 0.62) | 0.107 | -0.35(-0.74 – 0.04) | 0.077 |
|  | T/G | 48.40% |  |  |  |  |  |  |
|  | T/T | 20.64% |  |  |  |  |  |  |
