# Supplementary material for: Genetic variability in ADAM17/TACE is associated with sporadic Alzheimer’s disease risk, neuropsychiatric symptoms and cognitive performance on the Rey Auditory Verbal Learning and Clock Drawing Tests
Source: PLoS One. 2025 May 6;20(5):e0309631. doi: 10.1371/journal.pone.0309631 (PMC12054869; doi:10.1371/journal.pone.0309631)
Supplement: S5 Table — (DOCX) [file pone.0309631.s005.docx]

**S5 Table. Genotype distributions of the tag-SNPs and their associations with the Rey Auditory Verbal Learning Test – delayed recall score**

| **Tag-SNPs** | **Genotypes** | **sAD group** | **Genetic model** | | | | | |
| --- | --- | --- | --- | --- | --- | --- | --- | --- |
|  |  |  | **Additive** | | **Dominant** | | **Recessive** | |
|  |  |  | **Mean Difference (95% CI)** | **P-value** | **Mean Difference (95% CI)** | **P-value** | **Mean Difference (95% CI)** | **P-value** |
| **rs11690078** | T/T | 37.34% | -0.01(-0.24 – 0.22) | 0.912 | 0.09(-0.34 – 0.53) | 0.679 | -0.08(-0.42 – 0.25) | 0.632 |
|  | C/T | 46.84% |  |  |  |  |  |  |
|  | C/C | 15.82% |  |  |  |  |  |  |
| **rs35280016** | G/G | 61.48% | 0.1(-0.20 – 0.40) | 0.512 | 0.33(-0.56 – 1.22) | 0.471 | 0.08(-0.26 – 0.43) | 0.63 |
|  | A/G | 35.14% |  |  |  |  |  |  |
|  | A/A | 3.38% |  |  |  |  |  |  |
| **rs55694483** | A/A | 31.70% | -0.04(-0.28 – 0.20) | 0.727 | -0.07(-0.44 – 0.30) | 0.706 | -0.04(-0.47 – 0.39) | 0.856 |
|  | G/A | 49.30% |  |  |  |  |  |  |
|  | G/G | 19.00% |  |  |  |  |  |  |
| **rs12464398** | T/T | 45.85% | -0.14(-0.37 – 0.09) | 0.238 | -0.5(-0.98 – -0.02) | **0.041** | -0.05(-0.38 – 0.28) | 0.784 |
|  | T/C | 40.13% |  |  |  |  |  |  |
|  | C/C | 14.02% |  |  |  |  |  |  |
| **rs10179642** | T/T | 75.32% | 0.16(-0.20 – 0.52) | 0.394 | 0.92(-1.05 – 2.89) | 0.361 | 0.14(-0.24 – 0.51) | 0.474 |
|  | C/T | 24.05% |  |  |  |  |  |  |
|  | C/C | 0.63% |  |  |  |  |  |  |
|  | C/T | 46.46% |  |  |  |  |  |  |
|  | C/C | 10.32% |  |  |  |  |  |  |
| **rs13008101** | G/G | 30.96% | -0.26(-0.49 – -0.03) | **0.027** | -0.25(-0.61 – 0.11) | 0.167 | -0.46(-0.85 – -0.06) | 0.023 |
|  | T/G | 48.40% |  |  |  |  |  |  |
|  | T/T | 20.64% |  |  |  |  |  |  |
