# Supplementary material for: Genetic variability in ADAM17/TACE is associated with sporadic Alzheimer’s disease risk, neuropsychiatric symptoms and cognitive performance on the Rey Auditory Verbal Learning and Clock Drawing Tests
Source: PLoS One. 2025 May 6;20(5):e0309631. doi: 10.1371/journal.pone.0309631 (PMC12054869; doi:10.1371/journal.pone.0309631)
Supplement: S6 Table — (DOCX) [file pone.0309631.s006.docx]

**S6 Table. Genotype distributions of the tag-SNPs and their associations with the Babcock Story Recall Test score**

| **Tag-SNPs** | **Genotypes** | **sAD group** | **Genetic model** | | | | | |
| --- | --- | --- | --- | --- | --- | --- | --- | --- |
|  |  |  | **Additive** | | **Dominant** | | **Recessive** | |
|  |  |  | **Mean Difference (95% CI)** | **P-value** | **Mean Difference (95% CI)** | **P-value** | **Mean Difference (95% CI)** | **P-value** |
| **rs11690078** | T/T | 38.02% | -0.13(-0.36 – 0.11) | 0.283 | -0.13(-0.58 – 0.32) | 0.569 | -0.2(-0.54 – 0.15) | 0.262 |
|  | C/T | 45.78% |  |  |  |  |  |  |
|  | C/C | 16.20% |  |  |  |  |  |  |
| **rs35280016** | G/G | 62.23% | 0.32(0.01 – 0.62) | **0.043** | 0.84(-0.06 – 1.75) | 0.068 | 0.3(-0.06 – 0.66) | 0.102 |
|  | A/G | 31.85% |  |  |  |  |  |  |
|  | A/A | 5.92% |  |  |  |  |  |  |
| **rs55694483** | A/A | 31.81% | 0.27(0.03 – 0.51) | **0.026** | 0.36(-0.00 – 0.73) | 0.051 | 0.36(-0.06 – 0.79) | 0.094 |
|  | G/A | 47.74% |  |  |  |  |  |  |
|  | G/G | 20.45% |  |  |  |  |  |  |
| **rs12464398** | T/T | 47.14% | 0.13(-0.12 – 0.37) | 0.309 | 0.2(-0.32 – 0.71) | 0.45 | 0.15(-0.18 – 0.49) | 0.367 |
|  | T/C | 40.72% |  |  |  |  |  |  |
|  | C/C | 12.14% |  |  |  |  |  |  |
| **rs10179642** | T/T | 74.46% | -0.18(-0.55 – 0.18) | 0.324 | 0.82(-1.17 – 2.81) | 0.419 | -0.23(-0.61 – 0.15) | 0.235 |
|  | C/T | 24.84% |  |  |  |  |  |  |
|  | C/C | 0.70% |  |  |  |  |  |  |
| **rs12692385** | T/T | 43.48% | -0.22(-0.48 – 0.04) | 0.104 | -0.49(-1.06 – 0.09) | 0.097 | -0.2(-0.54 – 0.15) | 0.261 |
|  | C/T | 47.10% |  |  |  |  |  |  |
|  | C/C | 9.42% |  |  |  |  |  |  |
| **rs13008101** | G/G | 30.00% | -0.07(-0.30 – 0.17) | 0.572 | 0(-0.37 – 0.37) | 0.995 | -0.2(-0.61 – 0.21) | 0.329 |
|  | T/G | 21.42% |  |  |  |  |  |  |
|  | T/T | 48.58% |  |  |  |  |  |  |
