# Supplementary material for: Genetic variability in ADAM17/TACE is associated with sporadic Alzheimer’s disease risk, neuropsychiatric symptoms and cognitive performance on the Rey Auditory Verbal Learning and Clock Drawing Tests
Source: PLoS One. 2025 May 6;20(5):e0309631. doi: 10.1371/journal.pone.0309631 (PMC12054869; doi:10.1371/journal.pone.0309631)
Supplement: S7 Table — (DOCX) [file pone.0309631.s007.docx]

**S7 Table. Genotype distributions of the tag-SNPs and their associations with the Clock Drawing Test score**

| **Tag-SNPs** | **Genotypes** | **sAD group** | **Genetic model** | | | | | |
| --- | --- | --- | --- | --- | --- | --- | --- | --- |
|  |  |  | **Additive** | | **Dominant** | | **Recessive** | |
|  |  |  | **Mean Difference (95% CI)** | **P-value** | **Mean Difference (95% CI)** | **P-value** | **Mean Difference (95% CI)** | **P-value** |
| **rs11690078** | T/T | 37.50% | -0.24(-0.53 – 0.05) | 0.111 | -0.32(-0.87 – 0.22) | 0.248 | -0.32(-0.75 – 0.11) | 0.149 |
|  | C/T | 44.32% |  |  |  |  |  |  |
|  | C/C | 18.18% |  |  |  |  |  |  |
| **rs35280016** | G/G | 63.86% | -0.03(-0.43 – 0.37) | 0.882 | -0.12(-1.52 – 1.28) | 0.868 | - | - |
|  | A/G | 33.74% |  |  |  |  |  |  |
|  | A/A | 2.40% |  |  |  |  |  |  |
| **rs55694483** | A/A | 28.40% | 0.04(-0.28 – 0.36) | 0.81 | -0.16(-0.66 – 0.34) | 0.527 | 0.31(-0.23 – 0.86) | 0.263 |
|  | G/A | 50.62% |  |  |  |  |  |  |
|  | G/G | 20.98% |  |  |  |  |  |  |
| **rs12464398** | T/T | 49.42% | 0.06(-0.22 – 0.35) | 0.663 | -0.18(-0.75 – 0.39) | 0.538 | 0.23(-0.18 – 0.65) | 0.271 |
|  | T/C | 34.48% |  |  |  |  |  |  |
|  | C/C | 16.10% |  |  |  |  |  |  |
| **rs10179642** | T/T | 72.72% | 0.19(-0.25 – 0.63) | 0.404 | -0.1(-2.08 – 1.89) | 0.924 | - | - |
|  | C/T | 26.14% |  |  |  |  |  |  |
|  | C/C | 1.14% |  |  |  |  |  |  |
| **rs12692385** | T/T | 41.37% | -0.14(-0.46 – 0.18) | 0.4 | -0.23(-0.89 – 0.43) | 0.495 | -0.15(-0.58 – 0.28) | 0.49 |
|  | C/T | 47.13% |  |  |  |  |  |  |
|  | C/C | 11.50% |  |  |  |  |  |  |
