# Supplementary material for: Genetic variability in ADAM17/TACE is associated with sporadic Alzheimer’s disease risk, neuropsychiatric symptoms and cognitive performance on the Rey Auditory Verbal Learning and Clock Drawing Tests
Source: PLoS One. 2025 May 6;20(5):e0309631. doi: 10.1371/journal.pone.0309631 (PMC12054869; doi:10.1371/journal.pone.0309631)
Supplement: S8 Table — (DOCX) [file pone.0309631.s008.docx]

**S8 Table. Genotype distributions of the tag-SNPs and their associations with the Rey-Osterrieth complex figure copying test score**

| **Tag-SNPs** | **Genotypes** | **sAD group** | **Genetic model** | | | | | |
| --- | --- | --- | --- | --- | --- | --- | --- | --- |
|  |  |  | **Additive** | | **Dominant** | | **Recessive** | |
|  |  |  | **Mean Difference (95% CI)** | **P-value** | **Mean Difference (95% CI)** | **P-value** | **Mean Difference (95% CI)** | **P-value** |
| **rs11690078** | T/T | 41.18% | -0.38(-0.75 – -0.02) | **0.039** | -0.28(-1.01 – 0.44) | 0.439 | -0.67(-1.20 – -0.14) | **0.013** |
|  | C/T | 41.18% |  |  |  |  |  |  |
|  | C/C | 17.64% |  |  |  |  |  |  |
| **rs35280016** | G/G | 59.58% | 0.15(-0.40 – 0.69) | 0.596 | -0.89(-2.89 – 1.12) | 0.386 | - | - |
|  | A/G | 38.30% |  |  |  |  |  |  |
|  | A/A | 2.12% |  |  |  |  |  |  |
| **rs55694483** | A/A | 27.65% | -0.01(-0.46 – 0.43) | 0.948 | -0.27(-0.93 – 0.39) | 0.423 | 0.33(-0.45 – 1.12) | 0.401 |
|  | G/A | 55.33% |  |  |  |  |  |  |
|  | G/G | 17.02% |  |  |  |  |  |  |
| **rs12464398** | T/T | 40.00% | 0.01(-0.40 – 0.42) | 0.973 | -0.05(-0.87 – 0.76) | 0.898 | 0.04(-0.53 – 0.62) | 0.889 |
|  | T/C | 46.00% |  |  |  |  |  |  |
|  | C/C | 14.00% |  |  |  |  |  |  |
| **rs10179642** | T/T | 80.00% | -0.26(-0.97 – 0.44) | 0.46 | -0.26(-0.97 – 0.44) | 0.46 | - | - |
|  | C/T | 20.00% |  |  |  |  |  |  |
|  | C/C | 0% |  |  |  |  |  |  |
| **rs12692385** | T/T | 38.78% | 0.03(-0.39 – 0.45) | 0.89 | -0.11(-0.93 – 0.71) | 0.792 | 0.12(-0.47 – 0.70) | 0.7 |
|  | C/T | 46.94% |  |  |  |  |  |  |
|  | C/C | 14.28% |  |  |  |  |  |  |
| **rs13008101** | G/G | 26.54% | -0.19(-0.62 – 0.23) | 0.368 | -0.18(-0.83 – 0.46) | 0.574 | -0.34(-1.06 – 0.39) | 0.367 |
|  | T/G | 55.10% |  |  |  |  |  |  |
|  | T/T | 18.36% |  |  |  |  |  |  |
