# Supplementary material for: Genetic variability in ADAM17/TACE is associated with sporadic Alzheimer’s disease risk, neuropsychiatric symptoms and cognitive performance on the Rey Auditory Verbal Learning and Clock Drawing Tests
Source: PLoS One. 2025 May 6;20(5):e0309631. doi: 10.1371/journal.pone.0309631 (PMC12054869; doi:10.1371/journal.pone.0309631)
Supplement: S9 Table — (DOCX) [file pone.0309631.s009.docx]

**S9 Table.** **Genotype distributions of the tag-SNPs and their associations with the Attentive Matrices score**

| **Tag-SNPs** | **Genotypes** | **sAD group** | **Genetic model** | | | | | |
| --- | --- | --- | --- | --- | --- | --- | --- | --- |
|  |  |  | **Additive** | | **Dominant** | | **Recessive** | |
|  |  |  | **Mean Difference (95% CI)** | **P-value** | **Mean Difference (95% CI)** | **P-value** | **Mean Difference (95% CI)** | **P-value** |
| **rs11690078** | T/T | 36.98% | 0.1(-0.14 – 0.34) | 0.413 | 0.16(-0.29 – 0.62) | 0.484 | 0.11(-0.23 – 0.45) | 0.519 |
|  | C/T | 47.96% |  |  |  |  |  |  |
|  | C/C | 15.06% |  |  |  |  |  |  |
| **rs35280016** | G/G | 63.24% | 0.1(-0.22 – 0.41) | 0.546 | -0.08(-1.09 – 0.93) | 0.877 | - | - |
|  | A/G | 33.82% |  |  |  |  |  |  |
|  | A/A | 2.94% |  |  |  |  |  |  |
| **rs55694483** | A/A | 32.12% | -0.07(-0.31 – 0.17) | 0.562 | -0.06(-0.43 – 0.30) | 0.733 | -0.14(-0.57 – 0.29) | 0.527 |
|  | G/A | 48.18% |  |  |  |  |  |  |
|  | G/G | 19.70% |  |  |  |  |  |  |
| **rs12464398** | T/T | 44.88% | -0.09(-0.32 – 0.15) | 0.466 | -0.17(-0.64 – 0.30) | 0.485 | -0.09(-0.42 – 0.24) | 0.586 |
|  | T/C | 40.64% |  |  |  |  |  |  |
|  | C/C | 14.48% |  |  |  |  |  |  |
| **rs10179642** | T/T | 75.34% | 0.07(-0.30 – 0.43) | 0.711 | 0.08(-1.92 – 2.07) | 0.941 | - | - |
|  | C/T | 23.98% |  |  |  |  |  |  |
|  | C/C | 0.68% |  |  |  |  |  |  |
| **rs12692385** | T/T | 43.36% | -0.04(-0.30 – 0.21) | 0.749 | -0.14(-0.68 – 0.41) | 0.623 | -0.02(-0.36 – 0.32) | 0.904 |
|  | C/T | 46.16% |  |  |  |  |  |  |
|  | C/C | 10.48% |  |  |  |  |  |  |
| **rs13008101** | G/G | 32.16% | 0.02(-0.21 – 0.25) | 0.868 | 0.19(-0.16 – 0.55) | 0.28 | -0.2(-0.61 – 0.21) | 0.339 |
|  | T/G | 47.56% |  |  |  |  |  |  |
|  | T/T | 20.28% |  |  |  |  |  |  |
