# Supplementary material for: Genetic variability in ADAM17/TACE is associated with sporadic Alzheimer’s disease risk, neuropsychiatric symptoms and cognitive performance on the Rey Auditory Verbal Learning and Clock Drawing Tests
Source: PLoS One. 2025 May 6;20(5):e0309631. doi: 10.1371/journal.pone.0309631 (PMC12054869; doi:10.1371/journal.pone.0309631)
Supplement: S10 Table — (DOCX) [file pone.0309631.s010.docx]

**S10 Table. Genotype distributions of the tag-SNPs and their associations with the Verbal Fluency Test score**

| **Tag-SNPs** | **Genotypes** | **sAD group** | **Genetic model** | | | | | |
| --- | --- | --- | --- | --- | --- | --- | --- | --- |
|  |  |  | **Additive** | | **Dominant** | | **Recessive** | |
|  |  |  | **Mean Difference (95% CI)** | **P-value** | **Mean Difference (95% CI)** | **P-value** | **Mean Difference (95% CI)** | **P-value** |
| **rs11690078** | T/T | 38.20% | 0.03(-0.21 – 0.26) | 0.817 | 0.12(-0.33 – 0.57) | 0.613 | -0.01(-0.35 – 0.33) | 0.963 |
|  | C/T | 45.83% |  |  |  |  |  |  |
|  | C/C | 15.97% |  |  |  |  |  |  |
| **rs35280016** | G/G | 61.94% | 0.09(-0.22 – 0.40) | 0.576 | 0.35(-0.57 – 1.28) | 0.454 | - | - |
|  | A/G | 34.32% |  |  |  |  |  |  |
|  | A/A | 3.74% |  |  |  |  |  |  |
| **rs55694483** | A/A | 34.58% | 0(-0.24 – 0.24) | 0.985 | 0.12(-0.24 – 0.49) | 0.501 | -0.19(-0.63 – 0.25) | 0.393 |
|  | G/A | 46.62% |  |  |  |  |  |  |
|  | G/G | 18.80% |  |  |  |  |  |  |
| **rs12464398** | T/T | 45.46% | -0.04(-0.27 – 0.19) | 0.734 | -0.06(-0.52 – 0.40) | 0.811 | -0.05(-0.39 – 0.28) | 0.751 |
|  | T/C | 39.16% |  |  |  |  |  |  |
|  | C/C | 15.38% |  |  |  |  |  |  |
| **rs10179642** | T/T | 75.69% | 0(-0.37 – 0.37) | 0.994 | -0.73(-2.71 – 1.25) | 0.468 | - | - |
|  | C/T | 23.61% |  |  |  |  |  |  |
|  | C/C | 0.70% |  |  |  |  |  |  |
| **rs12692385** | T/T | 45.39% | 0.02(-0.23 – 0.27) | 0.87 | 0.2(-0.34 – 0.75) | 0.464 | -0.04(-0.38 – 0.30) | 0.814 |
|  | C/T | 43.97% |  |  |  |  |  |  |
|  | C/C | 10.64% |  |  |  |  |  |  |
| **rs13008101** | G/G | 33.33% | 0.04(-0.20 – 0.27) | 0.766 | 0.23(-0.13 – 0.58) | 0.208 | -0.2(-0.62 – 0.22) | 0.343 |
|  | T/G | 46.82% |  |  |  |  |  |  |
|  | T/T | 19.85% |  |  |  |  |  |  |
