# Supplementary material for: Genetic variability in ADAM17/TACE is associated with sporadic Alzheimer’s disease risk, neuropsychiatric symptoms and cognitive performance on the Rey Auditory Verbal Learning and Clock Drawing Tests
Source: PLoS One. 2025 May 6;20(5):e0309631. doi: 10.1371/journal.pone.0309631 (PMC12054869; doi:10.1371/journal.pone.0309631)
Supplement: S11 Table — (DOCX) [file pone.0309631.s011.docx]

**S11 Table. Genotype distributions of the tag-SNPs and their associations with the NPI Total Score**

| **Tag-SNPs** | **Genotypes** | **sAD group** | **Genetic model** | |  |  |  |  |  |
| --- | --- | --- | --- | --- | --- | --- | --- | --- | --- |
|  |  |  | **Additive** | | | **Dominant** | | **Recessive** | |
|  |  |  | **Mean Difference (95% CI)** | **P-value** | | **Mean Difference (95% CI)** | **P-value** | **Mean Difference (95% CI)** | **P-value** |
| **rs11690078** | T/T | 37.06% | 0.07(-2.10 – 2.24) | 0.951 | | 0.01(-3.15 – 3.17) | 0.993 | 0.22(-3.89 – 4.33) | 0.916 |
|  | C/T | 46.40% |  |  |  |  |  |  |  |
|  | C/C | 16.54% |  |  |  |  |  |  |  |
| **rs35280016** | G/G | 65.04% | -0.37(-3.13 – 2.39) | 0.793 | | -1.55(-4.87 – 1.77) | 0.361 | 5.33(-2.27 – 12.94) | 0.169 |
|  | A/G | 30.45% |  |  |  |  |  |  |  |
|  | A/A | 4.51% |  |  |  |  |  |  |  |
| **rs55694483** | A/A | 28.57% | -0.18(-2.39 – 2.02) | 0.871 | | 0.64(-2.77 – 4.04) | 0.714 | -1.33(-5.13 – 2.46) | 0.491 |
|  | G/A | 50.75% |  |  |  |  |  |  |  |
|  | G/G | 20.68% |  |  |  |  |  |  |  |
| **rs12464398** | T/T | 49.64% | 0.76(-1.45 – 2.97) | 0.502 | | 2.45(-0.60 – 5.51) | 0.115 | -2.31(-6.97 – 2.34) | 0.331 |
|  | T/C | 38.05% |  |  |  |  |  |  |  |
|  | C/C | 12.31% |  |  |  |  |  |  |  |
| **rs10179642** | T/T | 73.38% | 0.71(-2.46 – 3.88) | 0.66 | | 0.6(-2.85 – 4.05) | 0.733 | 3.3(-9.49 – 16.09) | 0.613 |
|  | C/T | 25.18% |  |  |  |  |  |  |  |
|  | C/C | 1.44% |  |  |  |  |  |  |  |
| **rs12692385** | T/T | 44.00% | -1.04(-3.34 – 1.27) | 0.379 | | -0.55(-3.63 – 2.54) | 0.728 | -3.29(-8.19 – 1.60) | 0.188 |
|  | C/T | 45.10% |  |  |  |  |  |  |  |
|  | C/C | 10.90% |  |  |  |  |  |  |  |
| **rs13008101** | G/G | 29.10% | -0.94(-3.13 – 1.26) | 0.402 | | 1.33(-2.06 – 4.71) | 0.442 | -4.47(-8.22 – -0.71) | **0.02** |
|  | T/G | 50.18% |  |  |  |  |  |  |  |
|  | T/T | 20.72% |  |  |  |  |  |  |  |
