# Supplementary material for: Genetic variability in ADAM17/TACE is associated with sporadic Alzheimer’s disease risk, neuropsychiatric symptoms and cognitive performance on the Rey Auditory Verbal Learning and Clock Drawing Tests
Source: PLoS One. 2025 May 6;20(5):e0309631. doi: 10.1371/journal.pone.0309631 (PMC12054869; doi:10.1371/journal.pone.0309631)
Supplement: S12 Table — (DOCX) [file pone.0309631.s012.docx]

**S12 Table.** **Genotype distributions of the tag-SNPs showing a nominal association with NPI subscores**

| **Tag-SNPs** | **Genotypes** | **sAD group** | **Genetic model** | | | | | |
| --- | --- | --- | --- | --- | --- | --- | --- | --- |
|  |  |  | **Additive** | | **Dominant** | | **Recessive** | |
|  |  |  | **Mean difference (95% CI)** | **P-value** | **Mean difference (95% CI)** | **P-value** | **Mean difference (95% CI)** | **P-value** |
|  | | | **Deliri** | | | | | |
| rs13008101 | G/G | 29.10% | 0.09(-0.23 – 0.41) | 0.593 | 0.5(0.01 – 0.99) | **0.045** | -0.37(-0.92 – 0.18) | **0.189** |
|  | T/G | 50.18% |  |  |  |  |  |  |
|  | T/T | 20.72% |  |  |  |  |  |  |
|  | | | **Allucinazioni** | | | | | |
| rs10179642 | T/T | 73.40% | 0.34 (-0.03 – 0.71) | 0.072 | 0.13 (-0.28 – 0.53) | 0.536 | 3.76 (2.33 – 5.18) | **<0.001** |
|  | C/T | 25.20% |  |  |  |  |  |  |
|  | C/C | 1.40% |  |  |  |  |  |  |
|  | | | **Agitazione** | | | | | |
| rs35280016 | G/G | 65.00% | 0.85 (0.14 – 1.55) | **0.018** | 0.54 (-0.31 – 1.40) | 0.212 | 3.61 (1.70 – 5.53) | **<0.001** |
|  | A/G | 30.50% |  |  |  |  |  |  |
|  | A/A | 4.50% |  |  |  |  |  |  |
|  | | | **Euforia** | | | | | |
| rs55694483 | A/A | 28.57% | 0.07(-0.00 – 0.14) | **0.062** | 0.06(-0.05 – 0.18) | 0.271 | 0.13(0.00 – 0.25) | **0.047** |
|  | G/A | 50.75% |  |  |  |  |  |  |
|  | G/G | 20.68% |  |  |  |  |  |  |
|  | | | **Irritabilità** | | | | | |
| rs12464398 | T/T | 49.64% | 0.47(-0.10 – 1.05) | 0.106 | 0.82(0.03 – 1.61) | **0.042** | 0.2(-1.02 – 1.41) | 0.751 |
|  | T/C | 38.04% |  |  |  |  |  |  |
|  | C/C | 12.32% |  |  |  |  |  |  |
|  | | | **Sonno** | | | | | |
| rs13008101 | G/G | 29.10% | -0.45(-0.92 – 0.01) | 0.058 | -0.24(-0.96 – 0.48) | 0.512 | -1.04(-1.85 – -0.24) | **0.011** |
|  | T/G | 50.18% |  |  |  |  |  |  |
|  | T/T | 20.72% |  |  |  |  |  |  |
|  | | | **Alimentazione** | | | | | |
| rs13008101 | G/G | 29.10% | -0.13(-0.53 – 0.27) | 0.529 | 0.27(-0.35 – 0.89) | 0.396 | -0.73(-1.42 – -0.03) | **0.04** |
|  | T/G | 50.18% |  |  |  |  |  |  |
|  | T/T | 20.72% |  |  |  |  |  |  |
